# The Relationship between Media Consumption and Health-Related Anxieties after the Fukushima Daiichi Nuclear Disaster

**DOI:** 10.1371/journal.pone.0065331

**Published:** 2013-08-14

**Authors:** Amina Sugimoto, Shuhei Nomura, Masaharu Tsubokura, Tomoko Matsumura, Kaori Muto, Mikiko Sato, Stuart Gilmour

**Affiliations:** 1 Department of Global Health Policy, Graduate School of Medicine, The University of Tokyo, Bunkyo-ku, Tokyo, Japan; 2 Division of Social Communication System for Advanced Clinical Research, The Institute of Medical Science, The University of Tokyo, Minato-ku, Tokyo, Japan; 3 Department of Public Policy, The Institute of Medical Science, The University of Tokyo, Minato-ku, Tokyo, Japan; UC Davis School of Medicine, United States of America

## Abstract

**Background:**

The Fukushima Daiichi nuclear disaster caused a global panic by a release of harmful radionuclides. In a disaster setting, misusage of contemporary media sources available today can lead to disseminated incorrect information and panic. The study aims to build a scale which examines associations between media and individual anxieties, and to propose effective media usages for future disaster management.

**Methods:**

The University of Tokyo collaborated with the Fukushima local government to conduct a radiation-health-seminar for a total of 1560 residents, at 12 different locations in Fukushima. A 13 item questionnaire collected once before and after a radiation-seminar was used on factor analysis to develop sub-scales for multiple regression models, to determine relationships between the sub-scales and media type consumed. A paired t–test was used to examine any changes in sub-scale of pre- and post-seminar scores.

**Results:**

Three sub-scales were revealed and were associated with different media types: was with rumors, while concern for the future was positively associated with regional-newspapers and negatively with national-newspapers. Anxiety about social-disruption was associated with radio. The seminar had a significant effect on anxiety reduction for all the three sub-scales.

**Conclusion:**

Different media types were associated with various heightened concerns, and that a radiation seminar was helpful to reduce anxieties in the post-disaster setting. By tailoring post-disaster messages via specific media types, i.e., radio, it may be possible to effectively convey important information, as well as to calm fears about particular elements of post-disaster recovery and to combat rumors.

## Introduction

Significant radiation-release events are rare and can cause elevated health risks over a wide area. The consequences of such events can include large scale evacuations, health testing, risk assessment and closure of businesses such as farms and fisheries, leading to social disruption on a wide scale and a significant level of social insecurity and fear amongst affected citizens [1]. Modern disaster settings can receive significant media coverage from a wide range of contemporary media sources [2], enabling people to obtain information of varying quality about the health and social consequences of such an event. This media coverage can be available to those directly affected by the radiation-release events, and offer risks and opportunities in disaster management in modern settings [3]. Unreliable or alarmist media coverage may lead to dissemination of incorrect information, panic and misconceptions about both disaster response and the health consequences of the accident; however, authoritative or highly trusted media sources may provide an ideal avenue for disaster responders to disseminate important public health and safety messages [4]. Therefore, understanding the relationship between collective anxiety over the impact of radiation on individuals and society and various types of mass media may serve those responsible for planning or implementing disaster responses [5]. This is especially important because lay understanding of radiation risk can vary widely from the understanding of experts responding to a disaster [6], and media reporting of radiation risk has been heavily criticized for both its poor scientific basis and alarmism [7], [8]

The Great East Japan Earthquake and subsequent nuclear reactor shutdown at Fukushima created just such a radiation-release incident. Though much information about the events of the reactor shutdown and subsequent radiation release remains unclear [9], the progress of the disaster and its consequences for local residents have been well documented [10], and included: evacuation of some residents near the nuclear plant; movement restrictions on other residents; cessation of local agricultural and outdoor laboring activities; and closure of local food suppliers. Coupled with the evacuation of residents affected by the tsunami, the disaster affected a large area and left many local residents uncertain about the risks to their own health, the security of their immediate area and the long-term impact on their health, fertility, work opportunities and future plans.

In order to help the local residents come to terms with their situation, the University of Tokyo, in collaboration with the Fukushima local government, offered radiation risk seminars to residents of the affected areas concurrently with health assessments, which included administration of a questionnaire covering basic health and social concerns, as well as media consumption information. The aim of this study is to understand the anxieties felt by the residents, and to explore the relationship between the dimensions of anxiety and types of mass media used by residents to obtain information about radiation risks. This information may be useful in planning post-disaster media responses and identifying the best channels through which information should be disseminated.

## Methods

### Subjects and questionnaire survey

From June 20^th^ to July 19^th^ 2011, a total of 1560 residents of Soma city, Fukushima Prefecture, participated in radiation information seminars at 12 different locations, including the city hall, hospitals and schools. Information about the seminars was publically disseminated at local schools and the city hall. All participants were recruited through voluntary participation. As part of the radiation seminar, participants were administered a questionnaire both before and after the seminar, to assess their level of concern regarding the effect of radiation on health and the extent of change due to the seminar. As part of the questionnaire, information on media consumption was also collected. Because the questionnaire was not administered as a health assessment tool, but as an evaluation tool for the seminars, no health or mental well-being questions were asked. The questionnaire contained the following three major components:

1. Demographic information: This included age, sex, educational background, occupation, personal and family history of cancer, residential area, and living status (e.g., number of child relatives).
2. Media consumption information: Asked on arrival at the seminar, this section provided questions concerning frequency and types of mass media used to obtain information about the nuclear disaster. Respondents were asked to indicate the weekly frequency with which they used the following media: TV news, TV tabloid shows, national newspapers, regional newspapers, radio news, radio tabloid shows, internet news, internet personal websites, rumors and notice boards. Frequency was rated as: “do not use”, “use once a week”, “use a few times a week”, and “use more than once a day.”
3. Information on anxiety about radiation risks: The questionnaire contained 14 items addressing fears about radiation and health, future social and economic prospects, and the current social and physical circumstances in which respondents lived. All but one question was measured on a five-point Likert scale, from *not concerned at all* (1 point) to *very concerned* (5 points). One of the questions (question 14) was open-ended. The participants answered these questions twice: once on arrival at the seminar, and again upon completion of the seminar.

In a few cases, people aged below 16 and over 70 completed the questionnaire. These responses were excluded from the data set, because of suspicion that people in these age groups might be rephrasing their answers to describe their opinions on family member's worries or concerns. Age was categorized into three groups: younger than 35, between 35 and 50, and older than 50. Educational background was categorized into three groups: middle/high school, college, and university and above. Similarly, occupation was categorized into five groups: outdoor, medical/educational/technological, housewife, unemployed and other. Area where the residents live was categorized in to three groups: mountain, seaside, and urban.

Approval for the study was granted by the ethics committees at the Institute of Medical Science, the University of Tokyo, and written informed consent was obtained from all the participants prior to commencement of the seminar.

### Statistical analysis

After excluding one open-ended question, all 13 Likert-scale questions about radiation-related anxiety were converted into a scale to measure radiation concerns suitable for analysis by media-usage. Sub-scales amongst the 13 questions were identified by analyzing all pre-seminar responses using factor analysis with a principal component factor extraction method and varimax rotation. The cut-off point for selecting factors was at the point of inflection of the scree plot. Accepted sub-scales were then assembled using two methods: naïve construction in which the individual variables were summed together to construct scale values, and weighted construction, in which the scales were composed as scores from the rotated factors. Post-seminar scales were also generated using the same method applied to the post-seminar questions. All subscales are referred to as factors in this study.

Cronbach's Alpha was used as an internal reliability test. External reliability of the questionnaire was evaluated to measure the extent to which distributions of scores of the pre- and post-seminar questionnaires were similar, using a test-retest analysis on the difference between pre- and post-seminar scores for all individuals. For this analysis the naïve factors were used, as the weighted factors have mean zero both before and after the seminar, by design. Because the radiation seminar was expected to change subject's responses, a mean change score of less than zero was expected in this analysis. However, seminar served to contaminate test-retest analysis, so test-retest validity cannot be definitively established in this study. For this reason, the scale developed, though expected to be appropriate for the regression analysis presented here, should not be considered a valid or reliable measure of anxiety or radiation-related health concerns without further research. The relationship between each factor and key demographic and media use variables was explored using one-way analysis of variance (ANOVA). Variables shown to be statistically significant (p<0.05) were then analyzed using a multiple regression model. Backwards stepwise model selection was used for model building and residual analysis was used to check the validity of the regression model. These models were conducted on the weighted factor scores, so that outcome variables used in each of the regressions were not correlated with each other. The possible confounders of age, sex and area were retained in the models regardless of their significance. Since almost all respondents used TV news as a common information source, it was excluded from the modeling processes. A separate regression analysis was conducted for each factor identified by factor analysis.

## Results

Of the 1560 seminar participants, 1298 (83%) gave consent to participate in the health survey. There were 1277 valid responses (completion of demographic items), giving a final response rate of 79%. Response rates at individual sites varied between 50 to 89%. After excluding the very young and old, 969 observations (62%) remained in the data set.

Table 1 summarizes the demographic characteristics of the study sample. The rate of missing responses was lower than 10% for all questions. Over 70% of the participants were female, the majority were aged above 50 (64%), and 63% had their last education in either middle or high school. About half of the participants (46%) had a personal or family history of cancer. A few people were living alone (9%). It also shows the proportion of respondents who use media more than once a week by media type. Over 95% answered to having used TV news as a common media source. All the media types, except for radio tabloid shows, internet news and internet personal websites, were used by more than 50% of people as a common information source.

**Table 1:** Respondent demographics and media consumption.

Variable	Respondents	Frequency (%)
Age		
Under 35	1014	115 (11)
Between 35 to 49		246 (24)
Over 49		653 (64)
Sex		
Male	1011	277 (27)
Female		734 (73)
Education		
Middle/High school	982	615 (63)
College		216 (22)
University and above		151 (15)
Occupation		
Outdoor	939	98 (9.96)
Medical/Education/Tech		184 (18.70)
Housewife		270 (27.44)
Unemployed		161 (16.36)
Other		271 (27.54)
Cancer History (personal or family)		
No	965	519 (54)
Yes		446 (46.22)
Living status*		
Alone	1014	89 (9)
With partner		702 (69)
With parents		366 (36)
With children		592 (58)
With grandchild		140 (14)
Total number of media types used		
0–3 types	1014	124 (12)
4–6 types		435 (43)
7–8 types		455 (45)
Area		
Mountain	1014	211 (21)
Seaside		233 (23)
Urban		570 (56)
Age		Mean/sd
Female	1014	52.82/12.57
Male		52.40/12.64
Combined		52.55/12.61
Media type		
TV news		968 (95)
TV tabloid shows		713 (70)
National newspapers		631 (62)
Regional newspapers		778 (77)
Radio news		531 (52)
Radio tabloid shows		319 (31)
Internet news		393 (39)
Internet personal websites		143 (14)
Rumors		670 (66)
Notice board		668 (66)

* Multiple answers possible for this question.

Factor analysis was conducted on the anxiety section of the questionnaire. After excluding those who did not fill in all 13 items from the data set, 87% of the 969 participants were eligible for this analysis.

Application of the scree plot criterion resulted in a three factor solution explaining 95% of the variance. Table 2 shows the loadings of each variable on the three factors generated from the factor analysis; the highest loading for each variable is indicated in bold. A variable was considered associated with a factor if its loading was above 0.4, and assigned to the highest-loaded factor where a conflict occurred. Three clear dimensions of radiation-related concern were identified: *radiation/health fears* (questions 1–5 and 9), *fears for the future* (question 6–8), and *fears about social disruption* (question 10–13).

**Table 2:** Factor loadings for pre-seminar variables.

Variables	Factor 1	Factor 2	Factor 3
Own health	0.55	0.27	0.29
Family health	0.59	0.20	0.34
Food safety	0.84	0.21	0.20
Water safety	0.81	0.25	0.20
Danger of being exposed to radiation in daily life	0.64	0.32	0.32
Effect on employment	0.30	0.77	0.16
Infertility	0.29	0.56	0.08
Effect on income	0.18	0.77	0.19
Radiation dose in the neighboring area	0.61	0.26	0.44
State response	0.42	0.25	0.44
How long this will last?	0.36	0.07	0.53
Future evacuation order	0.35	0.27	0.57
Media information	0.38	0.27	0.62

Cronbach's Alpha was calculated at 0.91 for factor 1, 0.82 for factor 2, and 0.78 for factor 3, indicating good internal reliability. Post-seminar scales were also generated using the same method applied to the post-seminar questions, and produced exactly the same set of factors with very similar numerical details. Correlation between pre- and post-seminar scores for the naïve summed factors ranged from 0.60 (factor 1) to 0.74 (factor 3), and all three factors showed a statistically significant decrease from pre- to post-seminar testing (paired T-test p-value<0.0001 in all cases). For all three factors, more than 80% of respondents' post-seminar scores were equal to or lower than their pre-seminar scores, suggesting a consistent downward shift in measures of anxiety that is likely to be due to the effect of the seminars themselves rather than a consistent bias in the testing mechanism, though this effect cannot be ruled out.

Multiple regression results for the relationship between the three factors and key demographic variables and media types are shown in Table 3 (factor 1), Table 4 (factor 2) and Table 5 (factor 3). The only type of media associated with radiation/health fears was “rumors.” Fears for the future (factor 2) were associated with national and regional newspapers, with regular use of national newspapers associated with a reduced level of fear on this factor, while use of regional newspapers was associated with heightened fears. Fears about social disruption were only associated with radio news.

**Table 3:** Multiple regression model of factor one against the media variables.

Variables	Coefficient (95% CI)	Standard Error	T statistic	P value
Intercept	−0.66 (−0.86 to −0.47)	0.10	−6.62	<0.001
Experimental variables:				
Rumors				
Do not use	Ref			
Use	0.22 (0.09 to 0.35)	0.07	3.35	<0.001
Confounders:				
Age				
Under 35	0.24 (0.05 to 0.43)	0.10	2.51	0.01
Between 35 to 49	0.22 (0.07 to 0.36)	0.07	2.96	0.003
Over 49	Ref			
Sex				
Female	0.30 (0.17 to 0.44)	0.07	4.49	<0.001
Male	Ref			
Cancer History				
Yes	0.08 (−0.05 to 0.20)	0.06	1.21	0.2
No	Ref			
Area				
Mountain	0.30 (0.12 to 0.48)	0.09	3.31	<0.001
Downtown	0.14 (−0.01 to 0.28)	0.07	1.82	0.07
Seaside	Ref			
Living status				
Living with grandchild	0.30 (0.12 to 0.49)	0.09	3.17	0.002
Not living with grandchild	Ref			

**Table 4:** Multiple regression model of factor two against the media variables.

Variables	Coefficient (95% CI)	Standard Error	T statistic	P value
Intercept	−0.62 (−0.87 to −0.37)	0.13	−4.91	<0.001
Experimental variables:				
National newspapers				
Do not use	Ref			
Use	−0.14 (−0.26 to −0.01)	0.06	−2.17	0.03
Regional newspapers				
Do not use	Ref			
Use	0.18 (0.04 to 0.32)	0.07	2.46	0.01
Confounders:				
Age				
Under 35	0.13 (−0.06 to 0.31)	0.10	1.33	0.2
Between 35 to 49	0.11 (−0.03 to 0.26)	0.07	1.53	0.1
Over 49	Ref			
Sex				
Male	0.00 (−0.14 to 0.15)	0.08	0.06	1.0
Female	Ref			
Education				
Middle/High school	0.31 (0.13 to 0.49)	0.09	3.43	<0.001
College	0.17 (−0.02 to 0.39)	0.10	1.82	0.07
University and above	Ref			
Occupation				
Outdoor	0.59 (0.35 to 0.83)	0.12	4.76	<0.001
Medical/Education/Tech	0.28 (0.02 to 0.39)	0.09	2.18	0.03
Housewife	Ref			
Unemployed	0.08 (−0.12 to 0.28)	0.10	0.79	0.4
Other	0.19 (0.02 to 0.35)	0.08	2.19	0.03
Living status				
Living with children	0.14 (0.01 to 0.27)	0.06	2.17	0.03
Not living with children	Ref			
Area				
Mountain	0.02 (−0.13 to 0.17)	0.08	0.26	0.8
Seaside	0.07 (−0.07 to 0.21)	0.07	0.95	0.3
Downtown	Ref			

**Table 5:** Multiple regression model of factor three against the media variable.

Variables	Coefficient (95% CI)	Standard Error	T statistic	P value
Intercept	−0.14 (−0.34 to 0.06)	0.10	−1.37	0.2
Experimental variables:				
Radio				
Do not use	Ref			
Use	0.16 (0.05 to 0.27)	0.05	2.96	0.003
Confounders:				
Age				
Under 35	−0.12 (−0.29 to 0.06)	0.09	−1.33	0.2
Between 35 to 49	−0.18 (−0.34 to −0.02)	0.08	−2.15	0.03
Over 49	Ref			
Sex				
Female	0.30 (0.19 to 0.42)	0.06	5.14	<0.001
Male	Ref			
Area				
Mountain	0.03 (−0.10 to 0.17)	0.07	0.48	0.6
Seaside	0.02 (−0.11 to 0.15)	0.07	0.29	0.8
Downtown	Ref			

## Discussion

This study aimed to examine associations between mass media consumption and health-related anxiety after a radiation-release accident. The exploratory factor analysis on both pre- and post-seminar questions led to a meaningful three factor solution which showed strong internal reliability and some evidence of test-retest validity.

Analysis of factor 1 showed that those who use rumors as a source of radiation information were more worried about the radiation effects on health than those who do not, while the multiple regression model of factor 2 suggests that users of national newspapers were less worried about prospects for future, and those who use regional newspapers more worried about their future prospects. This may be because regional papers present less authoritative information about radiation risks and government actions than national newspapers, which contain clearer lines of communication with government officials over current/prospective countermeasures against radiation. A strong correlation was also found between fears about social disruption (factor 3) and use of radio news, but this may reflect the nature of disaster planning in Japan: possession of a radio is considered a key element of an individual disaster preparation plan [11], and it could be that those more worried about social disruption due to disasters are more likely to both own and use a radio as part of their disaster planning.

This study's most serious limitation can be attributed to the nature of the seminars. Our ability to test the external (test-retest) reliability of the questionnaire was constrained by the effect of the health seminars: these were designed to influence participant's radiation fears, and due to this a simple change in mean might be expected from the seminars. However, because the effectiveness of the seminar was assessed simultaneously with the test-retest validity of the questionnaire itself, it is impossible to say whether the change in level observed in pre- and post-seminar scores reflects a true effect of the seminars or a consistent bias in the second administration of the questionnaire. It is unlikely; however, that poor test-retest reliability would be associated with a consistent reduction in statistically significant levels of anxiety across the three factors: the most likely effect of test-retest errors is to create a non-differential bias, with post-seminar scores distributed randomly relative to the pre-test scores. Given such an assumption, the magnitude, significance and consistent distribution of changes in the three factors between pre- and post-seminar assessments suggests that the changes in level observed were likely to be due to the seminar itself rather than validity problems in the questionnaire.

A second limitation is that the findings of the study represent only an aging and female-majority population in rural settings. In an urban setting, levels of concern over the three factors and the availability to mass media types listed on this study may be different.

Selection bias also limited the findings of the study. Seminar attendance was voluntary, and the participants may already have higher levels of radiation concerns than would be expected in a random sample of residents of the area.

Despite these limitations, this study may be useful for those planning or implementing a response to a radiation disaster of uncertain duration, harm and scale, giving insights into the relationship between radiation concerns and media consumption patterns. In the immediate post-disaster setting, as emergency response efforts begin, those disseminating information about the government response and the immediate and long-term health risks, should consider carefully the media to be used. This study shows that television of all kinds is a ubiquitous medium for the dissemination of information, and an ideal way to distribute basic health and safety information after a radiation release. Both television and internet usage do not show any particular pattern of association with particular concerns. However, efforts should be made to combat rumors, by which means erroneous information about the health effects may be transmitted. In addition, disaster responders should consider the role of regional newspapers, which may be harder to target and overlooked in favor of national newspapers that may have a closer relationship to government and health departments, and easier lines of communication. In this study, those with higher levels of concern about the future were more likely to read regional newspapers, suggesting that these newspapers may offer an avenue by which disaster responders can calm fears about the future more readily. This study also shows that fears about social disruption are related to use of a radio, and suggest that messages targeted to radio broadcasting may help to reduce such concerns.

Although the effectiveness of radiation health seminars could not be assessed directly through this study, due to the simultaneous assessment of seminar effect and test-retest validity, the evidence from the survey is suggestive of an effect of such seminars in reducing attendants' concerns about all dimensions of the disaster. Early and comprehensive provision of such seminars, in conjunction with assessments of individual radiation exposure risks, may be useful in both reducing anxiety, establishing trust at a local community level, and identifying those who are most concerned about the disaster's effects and the best means of delivering information to them.

Finally, this study found no strong evidence that social media were associated with heightened or reduced fears or concerns. This is surprising given the well-publicized role of social networking sites such as Twitter and Facebook in disaster response [12], [13]. However, the age of the sample and relatively low useage of such media (about 15%) suggests that this population's media preferences are not reflective of other, younger populations [14], and more research is needed before definitive conclusions can be drawn about the value of social networking sites as methods of information dissemination after a disaster.

This study found that there was a significant association between some types of media use and concerns about health and public order after the disaster. Media messages can be tailored to different audiences and delivery methods by disaster response planners to maximize the impact of messages being delivered, to reduce anxiety, and to improve their ability to deliver correct, accurate and timely information in a post-disaster setting. Radiation health seminars may also provide a useful forum both for delivering health-related information and for assessing the best methods for disseminating post-disaster messages. Disaster planners should be aware that media consumption patterns are not homogeneous and attention to their heterogeneity may make post-disaster information dissemination more effective and responsive to public health needs.
